# Emergence and genomic analysis of a novel sublineage of bovine ephemeral fever virus in Southwest China

**DOI:** 10.3389/fmicb.2023.1161287

**Published:** 2023-03-22

**Authors:** Jing Chen, Mengru Liu, Yixuan Li, Liu Yang, Yunhan Tang, Ruitong Dan, Muhan Xie, Rendong Fang, Nengzhang Li, Chao Ye, Yuanyi Peng

**Affiliations:** College of Veterinary Medicine, Southwest University, Chongqing, China

**Keywords:** bovine ephemeral fever virus, genomic analysis, phylogenetic analysis, sublineage, recombination

## Abstract

**Introduction:**

Bovine ephemeral fever virus (BEFV), belonging to the genus *Ephemerovirus* under the family *Rhabdoviridae*, is the etiological cause for the bovine ephemeral fever (BEF) in cattle and water buffalo.

**Methods:**

In this study, we report recent BEF outbreaks in Southwest China and sequence the complete genome sequence of one BEFV isolate BEFV/CQ1/2022.

**Results and Discussion:**

Comparative genomic analyses between BEFV/CQ1/2022 and isolates available in GenBank revealed remarkable inter-isolate divergence. Meanwhile, the sequence divergence was related to the evolutionary relationships and geographical distribution of the isolates. Phylogenetic analysis indicated that the global BEFV isolates can be divided into 4 distinct lineages. The East Asia lineage was the most diverse and could be subdivided into 4 sublineages. Notably, BEFV/CQ1/2022 and other 10 recent isolates from Mainland China were found to be clustered in sublineage 2. Additionally, recombination analysis provided evidence of BEFV recombination among East Asian isolates for the first time. Taken together, a novel sublineage of the East Asian BEFV emerged in Southwest China, and large divergence and potential recombination among BEFV strains were investigated in this study, which may improve understanding of BEFV epidemiology and evolution.

## 1. Introduction

Bovine ephemeral fever virus (BEFV) is an arthropod-borne rhabdovirus under the genus *Ephemerovirus* in the *Rhabdoviridae* (Murphy et al., 1972). Bovine ephemeral fever virus is considered to be the etiological cause for bovine ephemeral fever (BEF) of cattle and water buffaloes in tropical and subtropical regions including Africa, Asia, the Middle East, and Australia (Zheng and Qiu, 2012; Trinidad et al., 2014). Bovine ephemeral fever occurs seasonally in adjacent temperate zones and mosquitoes act as major vectors for BEFV spreading (Trinidad et al., 2014). The disease is characterized by high fever (triphasic), pneumonia, depression, stiffness, lameness, and paralysis. Although the BEF case mortality is thought to be very low, morbidity rate may go as high as 100% (Pyasi et al., 2020).

Bovine ephemeral fever virus is an enveloped virus with a bullet-like shape, which is structurally similar to other mammalian rhabdoviruses such as rabies virus and vesicular stomatitis virus (Walker and Klement, 2015; Abayli et al., 2017). The genome of BEFV is a negative-sense single-stranded RNA with approximately 14.9 kb in length, mainly encoding 5 canonical rhabdovirus structural proteins in the following order: nucleoprotein (N), phosphoprotein (P), matrix protein (M), glycoprotein (G), and RNA-dependent RNA polymerase (L; Walker, 2005). The N protein is the most abundant element of virion that tightly associates with the genome, the P protein is essential for viral genome transcription and replication, the M protein encapsulates the nucleocapsid, the L protein is an RNA-dependent RNA polymerase that helps in the formation of transcription-replication complex, and the glycoprotein G is the only protein exposed to host cell surface during infection and acts as the major neutralizing antigen (Walker et al., 1991, 1992). Furthermore, a total of 4 independent antigenic sites (G1-G4) in the neutralizing G protein ectodomain have been identified. G1 (aa 487–503) is a linear antigenic site, G2 (aa 168–189) and G3 (aa 49–63, aa 215–231, and aa 262–271) are nonlinear conformational sites, however, the location of G4 has not yet been determined (Trinidad et al., 2014). In addition, a nonstructural glycoprotein (GNS) and several small accessory proteins (α1, α2, α3, β, and γ) are also located in the viral genome region between the G and L genes, but their functions are largely unknown (Walker, 2005).

Although the BEFV isolates are considered to be serologically related within a single cross-neutralizing serotype worldwide (Trinidad et al., 2014), phylogenetic analysis based on G gene sequences shows that the BEFV isolates worldwide can be classified into four different groups including the Australian, East Asian, Middle East and the relatively separate South African lineages (Kato et al., 2009; Zheng and Qiu, 2012; Omar et al., 2020). In China, BEF has been found endemic in Taiwan and 26 provinces in Mainland China since the first report in 1955 (Gao et al., 2017), and the first BEFV strain JB76H was isolated from an infected cow in Beijing in 1976 (Bai et al., 1987). Then, frequent epidemics of BEFV have been observed in Mainland China during the last two decades. For instance, the strain JT02L was isolated from dairy cattle in Zhejiang in southeast China in 2002 (Zheng et al., 2009), the LS11 and LYC11 strains were originated from dairy cattle in Henan in central China in 2011 (Zheng and Qiu, 2012), the 2011-Shandong isolate was from a cow with high fever in Shandong in eastern China in 2011 (Hou et al., 2012), and several isolates were collected in Guangdong and Guangxi provinces in Southern China in 2013–2017 (Kun et al., 2020). However, information on the nucleotide sequence variation of the BEFV isolates in Southwest China is still lacking.

In this study, we recently collected and analyzed clinical samples of BEF suspected cattle from outbreaks in Southwest China. Firstly, the complete G gene sequences of 4 samples from 3 different regions were obtained and they shared 100% homology. Then, the genome sequence of one sample (BEFV/CQ1/2022) was obtained by overlapping primer-based PCR and Sanger sequencing. Phylogenetic analysis based on BEFV complete sequences indicated that the current isolate BEFV/CQ1/2022 and one isolate BA/RZ/IR from Iran clustered within a sublineage of the East Asia lineage. Comparative genomic analysis suggested that large diversity existed among BEFV isolates with BEFV/CQ1/2022 highly similar to the East Asian strain JT02L but significantly different from isolates from other lineages. A phylogenetic tree based on G ectodomain encoding sequences indicated that the global BEFV isolates can be divided into 4 distinct lineages, and highlighted that the East Asia lineage was the most diverse and could be subdivided into four sublineages. Notably, BEFV/CQ1/2022 and other 10 recent isolates from Mainland China were clustered in the distinct sublineage 2. Recombination analysis showed that JT02L might be a minor parent-like strain of the Iranian strain BA/RZ/IR and BEFV/CQ1/2022 was more likely to be the major parent-associated strain with its ancestral strain as the actual major parent-like strain.

## 2. Materials and methods

### 2.1. Sample collection

In September 2022, the suspected outbreaks of bovine ephemeral fever in cattle occurred in Chongqing and Sichuan in Southwest China. With the owners’ consent, clinical samples including blood, nasal swab and several tissues from the symptomatic cattle (Table 1) were collected with sterile collection tubes and immediately transported to our laboratory at temperatures between 2 and 8°C for routine diagnostic purposes. Our sampling procedures were approved by Institutional Animal Care and Use Committee of Southwest University, Chongqing, China (IACUC-20221114-03).

**Table 1:** Clinical sample information in this study.

Samples No.	Collection date	Source	Geographical origin	PCR test results of BEFV
1	2022.9.14	Blood	Dianjiang, Chongqing	+ (test with pooled samples 1–3)
2	2022.9.14	Blood	Dianjiang, Chongqing	
3	2022.9.14	Blood	Dianjiang, Chongqing	
4	2022.9.14	Blood	Dianjiang, Chongqing	+ (test with pooled samples 4–6)
5	2022.9.14	Blood	Dianjiang, Chongqing	
6	2022.9.14	Blood	Dianjiang, Chongqing	
7	2022.9.14	Blood	Dianjiang, Chongqing	+ (test with pooled samples 7–9)
8	2022.9.14	Blood	Dianjiang, Chongqing	
9	2022.9.14	Blood	Dianjiang, Chongqing	
10	2022.9.14	Blood	Dianjiang, Chongqing	+ (test with pooled samples 10–12)
11	2022.9.14	Blood	Dianjiang, Chongqing	
12	2022.9.14	Blood	Dianjiang, Chongqing	
13	2022.9.14	Nasal swab	Dianjiang, Chongqing	+ (test with pooled samples 13–16)
14	2022.9.14	Nasal swab	Dianjiang, Chongqing	
15	2022.9.14	Nasal swab	Dianjiang, Chongqing	
16	2022.9.14	Nasal swab	Dianjiang, Chongqing	
17	2022.9.14	Nasal swab	Dianjiang, Chongqing	+ (test with pooled samples 17–18)
18	2022.9.14	Nasal swab	Dianjiang, Chongqing	
19	2022.9.14	Nasal swab	Dianjiang, Chongqing	+ (test with pooled samples 19–21)
20	2022.9.14	Nasal swab	Dianjiang, Chongqing	
21	2022.9.14	Nasal swab	Dianjiang, Chongqing	
22	2022.9.24	Lung	Fengdu, Chongqing	+
23	2022.9.24	Liver	Fengdu, Chongqing	−
24	2022.9.24	Spleen	Fengdu, Chongqing	−
25	2022.9.24	Kidney	Fengdu, Chongqing	−
26	2022.9.24	Heart	Fengdu, Chongqing	−
27	2022.9.24	Small intestine	Fengdu, Chongqing	−
28	2022.10.17	Lung	Meishan, Sichuan	+
29	2022.10.17	Liver	Meishan, Sichuan	−
30	2022.10.17	Spleen	Meishan, Sichuan	−
31	2022.10.17	Small intestine	Meishan, Sichuan	−
32	2022.10.17	Heart	Meishan, Sichuan	−

### 2.2. Etiological examinations of clinical samples

For etiological examinations, samples were either pooled or prepared separately, and then used for total RNA extraction by TRIzol reagent (Invitrogen, United States) according to the manufacturer’s instructions. RNA samples were then subjected to cDNA synthesis using PrimeScript^™^RT reagent Kit with gDNA Eraser (TaKaRa, Dalian, China). And the primers BEFV-F (AGAGCTTGGTGTGAATAC) and BEFV-R (CCAACCTACAACAGCAGATA) were used to examine the presence of BEFV.

### 2.3. Genome sequencing of BEFV

For both amplifying the complete genome sequence and the G gene of BEFV, a set of primers (shown in Table 2) based on conserved regions of the viral genome was designed and used for obtaining the BEFV whole genome (Primer pairs 1–9) and the G gene sequences (Primer pairs 2–3) in BEFV-positive samples. The RT-PCR amplicons of expected sizes were extracted from agarose gels, cloned into the pMD-19 T vector (TaKaRa, Dalian, China), and sequenced by Sanger sequencing using the universal primers M13-47/M13-48. The complete genome and the G gene sequences were then assembled using SeqMan software (DNASTAR, Madison, WI, United States). Subsequently, the whole genomic cDNA sequences of BEFV/CQ1/2022 were submitted to NCBI GenBank database with accession no. OP887034.

**Table 2:** Primers used for Bovine ephemeral fever virus (BEFV) genome amplification in this study.

Primer pairs	Sequence (5′–3′)	Locations^a^	Length of amplicon (bp)
1	ACGAGAAAAAACAAAAAAACTAATTGATA	1–29	2,728
	GCAGTTCCGGTGAATTCTATTACCTCG	2,702–2,728	
2	TTCAAAACCAATAGAAAGAACAAC	2,522–2,545	1,698
	TAAGATCCGATCCCATAATGAT	4,198–4,219	
3	ACAGATAGAACAGAATTTGAAG	3,921–3,942	1754
	GCTTAATCAACTCTAGTCTAAT	5,653–5,674	
4	ATTATCCTCCTCCAAAGTGCGA	5,393–5,414	1,560
	TCAATATAATCCAAAATCCTAG	6,931–6,952	
5	AACAGGCAATGGAGAAAGG	6,815–6,833	2,368
	GTTTGAAACCTGCTAATTA	9,164–9,182	
6	GGACATAAGTGGACGGTGGATA	9,063–9,084	1967
	TTTCTCCTCTGATTGCTGGATT	11,008–11,029	
7	GGAACAATAAAGGGACTGCCAA	10,807–10,828	2018
	GTGAACTCTGATATAAATGATG	12,803–12,824	
8	GGTAGGTGATTCACTGTTAAGT	12,582–12,603	1,375
	AGCATGTCTAATATATTTGTA	13,935–13,956	
9	GAAACAGAATTCTATGATGATTGACCTGAT	13,767–13,788	1,133
	ACGAAGAAAAACAAATAAAATACAATTCCT	14,870–14,899	

a The location is determined based on the representative strain Bovine/China/Henan1/2012 (KM276084.1).

### 2.4. Sequence annotation and protein-coding sequence alignments

The annotation of BEFV/CQ1/2022 was performed based on those annotations of the BEFV complete sequences available in the GenBank database. Then, the BEFV/CQ1/2022 genome was aligned with those of the representative isolates BA/RZ/IR (GenBank Accession No. MZ687779), JT02L (GenBank Accession No. KY315724), IND/IDR/BEFV/2019 (GenBank Accession No. MN905763), BB7721 (GenBank Accession No. AF234533) and RSA/OBP/BEF2008 (GenBank Accession No. MW463337). The multiple alignment was performed using the mVista LAGAN genomics analysis tool (Frazer et al., 2004). To investigate the diversity of each protein-coding sequences between isolates from different phylogenetic clusters, the corresponding amino acid alignments with the representative isolates (BEFV/CQ1/2022, JT02L, IND/IDR/BEFV/2019, BB7721 and RSA/OBP/BEF2008) were generated using MEGA 5.2 for sequence comparisons. Then, the amino acid variations unique to the corresponding sites of the BEFV/CQ1/2022 genome were identified and recorded in Table S1.

### 2.5. Phylogenetic analysis

The genomic sequence of BEFV isolate BEFV/CQ1/2022 was aligned with BEFV isolates available in the GenBank database using the MAFFT online server to investigate the genetic variation among BEFV isolates. Then, a phylogenetic tree was constructed using the MEGA 5.2 software and the maximum likelihood (ML) method and the Tamura-Nei model of nucleotide substitution with 1,000 bootstrap replications (Tamura et al., 2011).

In addition, the ectodomain encoding sequence of G gene was most widely used for the phylogeny of BEFV. To understand the genetic diversity of BEFV worldwide, all the available G ectodomain encoding sequences (1,527 nt) were retrieved from NCBI GenBank database and analyzed together with the corresponding sequence of BEFV/CQ1/2022 collected in this study. Multiple sequence alignment was performed using the MUSCLE program implemented within the MEGA 5.2 software. Phylogenetic analysis based on the alignment of these G ectodomain encoding sequences were performed in MEGA5.2. A phylogeny was constructed by the neighbor-joining (NJ) method with the Kimura 2-parameter model. The reliability was evaluated by the bootstrap test with 1,000 replicates.

### 2.6. Antigenic sites analysis of BEFV G protein

In this study, the aa sequences deduced from BEFV G ectodomain encoding sequences were aligned in the MEGA 5.2 software, and the aa sequence variation within the G1, G2 and G3 sites were analyzed with the DNASTAR MegAlign software. The representative BEFV isolates were selected according to their positions in the phylogenetic tree based on G ectodomain encoding sequences.

### 2.7. Recombination analysis

All the 16 complete sequences of BEFV in this study were aligned using the MAFFT online server with the default parameter settings, then analyzed using two different types of software. Firstly, the RDP4.39 software was used to detect potential inter-isolate recombination events and the positions of breakpoints. Default parameter settings were used and the threshold value of p was set at 0.05 using the Bonferroni correction. To further validate the putative recombination events, the SimPlot 3.5.1 program was then used to identify the putative recombinant, which performed the bootscan analysis with a sliding window of 500 bp, a step size of 10 bp, gap stripped, and the Kimura 2-parameter substitution model. Phylogenetic trees were then constructed to validate the detected recombination signals when querying strain BA/RZ/IR, using the alignments of recombination region and non-recombination region divided by the predicted breakpoints, respectively.

## 3. Results

### 3.1. Etiological analysis of clinical samples

In this study, a total of 32 clinical samples including 12 blood samples, 9 nasal swabs, and 11 tissue samples were collected and used for RNA extraction and cDNA synthesis. Then, the cDNAs of 12 blood samples and 9 nasal swabs obtained from Dianjiang in Chongqing city were pooled by 2–4 samples for the subsequent BEFV detection. Finally, the results of PCR detection showed that all the tested samples were BEFV positive (Table 1). Meanwhile, the lung samples collected from Fengdu in Chongqing city and Meishan in Sichuan province were also tested to be BEFV positive by PCR, respectively. But the liver, spleen, kidney, heart and small intestine were found to be BEFV negative (Table 1). Furthermore, 4 samples collected from Dianjiang (2 blood samples), Fengdu (1 lung sample), and Meishan (1 lung sample) were selected to obtain their G gene sequences by PCR and Sanger sequencing. The results showed that all the 4 samples shared 100% sequence homology (Data not shown), indicating that the recent BEF outbreaks in Southwest China may be caused by the same BEFV strain.

### 3.2. Comparative genomic analysis of BEFV isolates

**Figure 1:**
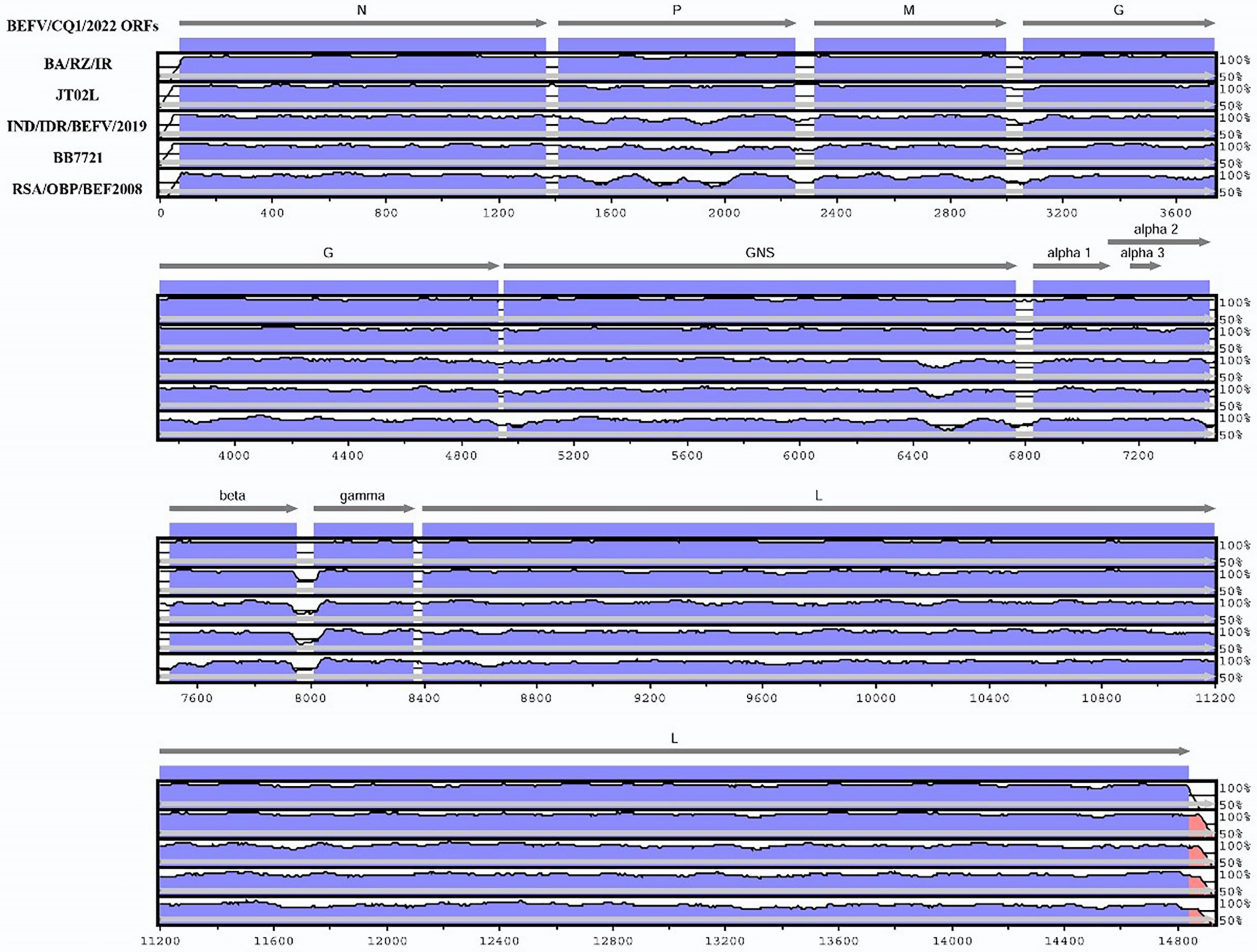
Genomic organization of Bovine ephemeral fever virus (BEFV) and comparison of sequence conservation within the BEFV/CQ1/2022, BA/RZ/IR, JT02L, IND/IDR/BEFV/2019, BB7721 and RSA/OBP/BEF2008 isolates. The mVISTA similarity plot showed sequence conservation among BEFV isolates BEFV/CQ1/2022, BA/RZ/IR, JT02L, IND/IDR/BEFV/2019, BB7721, and RSA/OBP/BEF2008. Sequence conservation was determined from a multiple sequence alignment, and the conservation score was plotted in a sliding 100-bp window.

**Figure 2:**
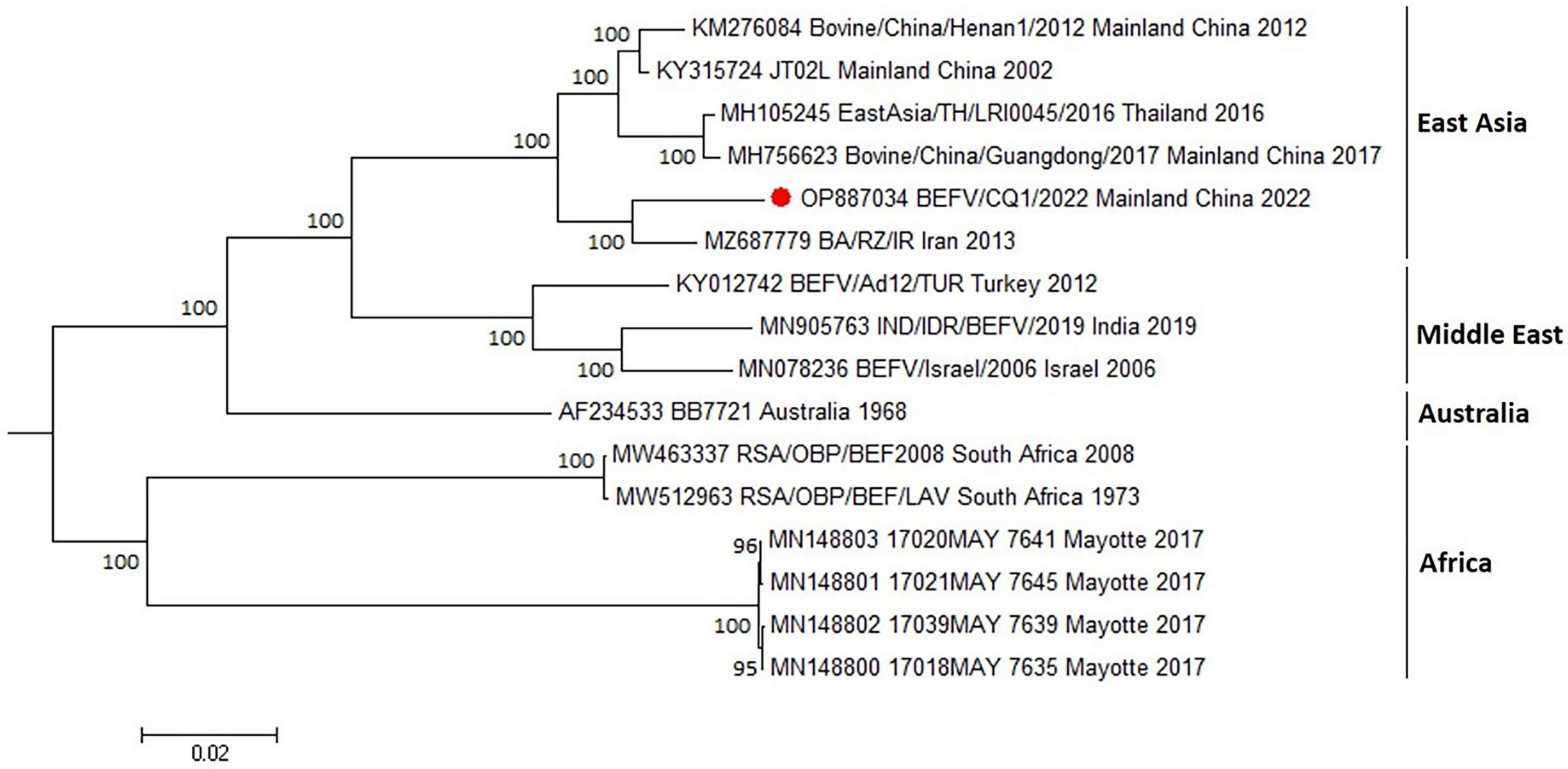
Phylogenetic tree construction based on the complete genome sequence of BEFV. The maximum likelihood (ML) method with bootstrap values (1,000 replicates) was used for phylogenetic tree construction with bootstrap values shown at the nodes. Bootstrap values less than 60% were not shown on the corresponding nodes. Red circle indicated the sample investigated in the current study. GenBank accession numbers along with isolates names, sample collection sites and year of the collection were indicated for reference viruses.

To investigate the genomic characterization of the BEFV isolate BEFV/CQ1/2022, the genome of BEFV/CQ1/2022 was sequenced and found to be 14,925 bp in length. Moreover, the BEFV/CQ1/2022 genome has the same genomic composition as the other strains studied, however, several hypervariable regions in the P, M, G, GNS, and L genes were found in the multi-genome alignment of the BEFV strains (Figure 1). Compared to the relative diversity found between BEFV/CQ1/2022 and IND/IDR/BEFV/2019, BB7721 & RSA/OBP/BEF2008 isolates, the genomic diversity between the isolates (BEFV/CQ1/2022, BA/RZ/IR and JT02L) showed fewer differences (Figure 1). Furthermore, the similarities in their genomic sequences were generally consistent with their phylogenetic relationships. Phylogenetic analysis indicated that IND/IDR/BEFV/2019, BB7721, RSA/OBP/BEF2008 and JT02L clustered in the lineages of Middle East, Australia, Africa and East Asia, respectively (Figure 2). Meanwhile, the BEFV/CQ1/2022 and BA/RZ/IR isolates were closely related and clustered in a sublineage of the East Asia lineage (Figure 2). In addition, numerous aa mutations were found between BEFV/CQ1/2022 and the isolates from the Middle East, Australia and Africa lineages (Table S1). By contrast, the East Asian isolates BEFV/CQ1/2022 and JT02L showed a high degree of identity in their protein coding sequences (Table S1). Furthermore, due to the frequent occurrence of initiation and termination codons mutations, several aa insertions or deletions were found in BEFV-encoded proteins. Compared to BEFV/CQ1/2022, the α2 ORF of JT02L, the α3 ORF of IND/IDR/BEFV/2019, the β ORF of BB7721, the GNS ORF of RSA/OBP/BEF2008, and the L ORF of JT02L, IND/IDR/BEFV/2019 and BB7721 were truncated by 4 aa (MFGY), 20 aa (KIGHNAKRRSKFRLLSVAQH), 40 aa (EEYGVIDISIKVEPRGLRFLKRSSEIDICDIP RKVRVVPT), 8 aa (QRFFKLDY) and 3 aa (MKK), respectively; meanwhile, the α3 ORF of JT02L and BB7721 were elongated by 22 aa (QRELVMFGRCKETDINRVPESF) and 16 aa (QRELVVSGGHEKTNIN), respectively; additionally, the start codon alteration (ATG to GTG) was found in the α3 ORF of RSA/OBP/BEF2008, which might cause a translation defect in the α3 ORF of this BEFV isolate (Table S1).

### 3.3. Phylogeny of BEFVs based on G ectodomain encoding sequences

**Figure 3:**
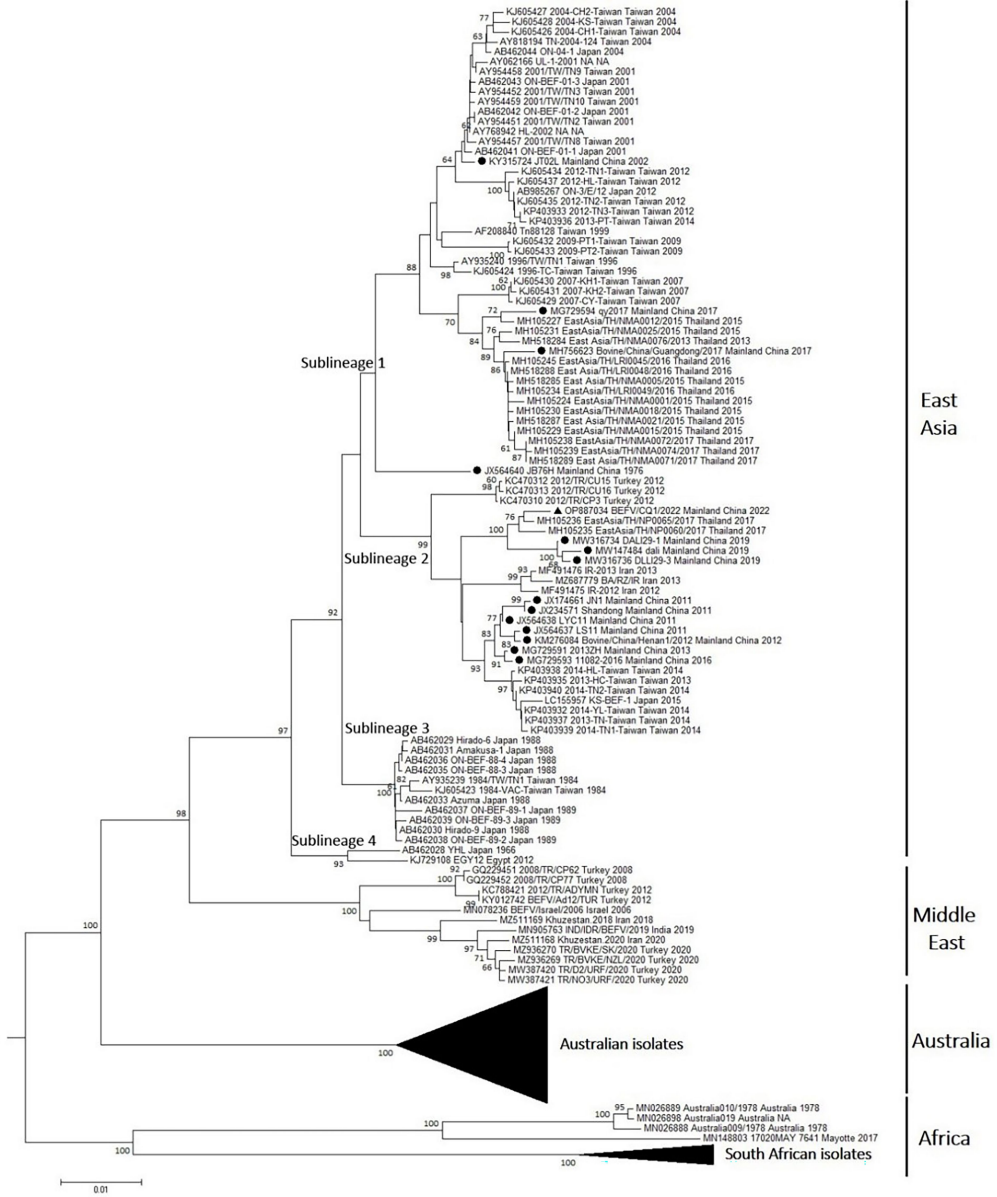
Phylogenetic tree of the BEFV isolates based on the comparison of G ectodomain encoding sequences. The phylogenetic tree of G ectodomain encoding sequences constructed by using neighbor-joining (NJ) method with Kimura 2-parameter model with bootstrap values of 1,000 replicates. The BEFV isolate BEFV/CQ1/2022 was indicated by black triangle, and other 14 BEFV strains from Mainland China were indicated by black circles. The details of Australian isolates in the Australia lineage and South African isolates in Africa lineage were not shown.

To predict the evolution of BEFVs worldwide, the 1,527 nt G ectodomain encoding sequence of BEFV/CQ1/2022 was aligned to the global data set deposited in the GenBank database. Then, the phylogenetic tree was constructed by the neighbor-joining (NJ) method. It showed that the global BEFV isolates were generally clustered geographically and divided into 4 large and distinct lineages (Figure 3). The East Asia lineage showed the greatest diversity and mostly consisted of isolates from Japan, Thailand, Mainland China and Taiwan with a few isolates from Turkey (3), Iran (3) and Egypt (1). By contrast, the Middle East lineage comprised isolates mostly from Middle East countries Turkey, Israel and Iran with only one isolate from India. Similarly, strains of the Australia lineage were all isolated in Australia. Additionally, the Africa lineage was composed of Australian and African isolates (Figure 3). Noticeably, the East Asia lineage could be subdivided into 4 sublineages (sublineage 1–4). Meanwhile, most of the isolates from Mainland China including BEFV/CQ1/2022 were found in sublineage 2, and the Middle East-derived isolates were found in sublineages 2 and 4 (Figure 3), suggesting a close evolutionary relationship between the recent East Asian isolates and Middle Eastern isolates.

### 3.4. Amino acid variation of the antigenic sites in BEFV G protein

**Figure 4:**
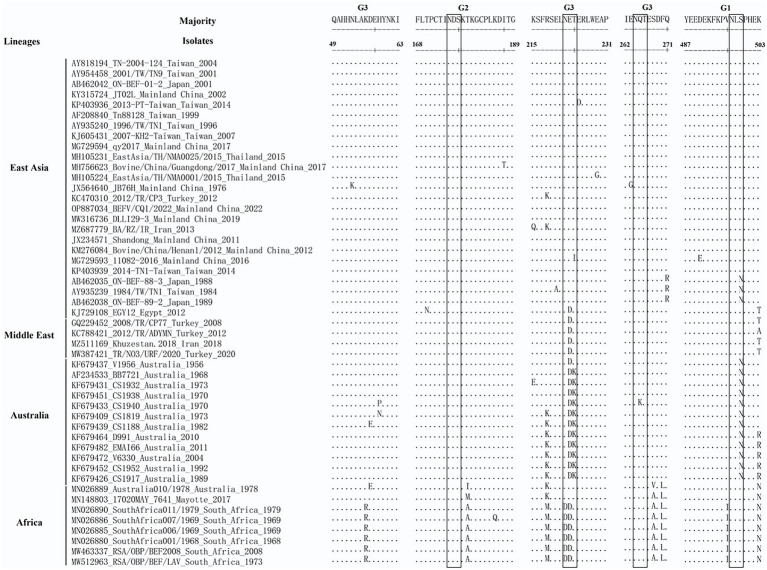
Comparison of antigenic sites of G protein among BEFV isolates. The amino acid residues differing from the sequences of the majority are denoted. Putative glycosylation sites are boxed.

As shown in Figure 4, the aa sequences of G1-G3 were relatively conserved among the BEFV isolates in the East Asia lineage, except for 15 aa substitutions in 11 isolates. Specifically, three residues at positions 490, 499, and 503 in the G1 sites were substituted from D to E, from S to N, and from K to T, respectively. And only two residues at positions 170 (T) and 187 (I) in the G2 sites were replaced with N and T, respectively. By contrast, a total of 10 substitutions (N53K, K215Q, R218K, E220A, E223D, T224I, E225D, E229G, E263G and Q271R) were found in the G3 sites (Figure 4). Additionally, compared to isolates from East Asia lineage, the aa sequences of G1-G3 among Middle East, Australia and Africa were significantly different. Most isolates from these lineages had their representative aa sequences in the antigenic sites, such as E223D and K503T in the Middle East lineage, R218K, E223D, T224K, S499N, K503R in the Australia lineage, K56R, T179A, R218M, N222D, E223D, S268A, F270L, V496I, K503N in the Africa lineage. In particular, some of the representative aa sequences were located at the putative glycosylation sites of G protein, which might influence the G protein antigenicity of isolates from these lineages (Figure 4).

### 3.5. Recombination analysis in BEFV isolates

**Figure 5:**
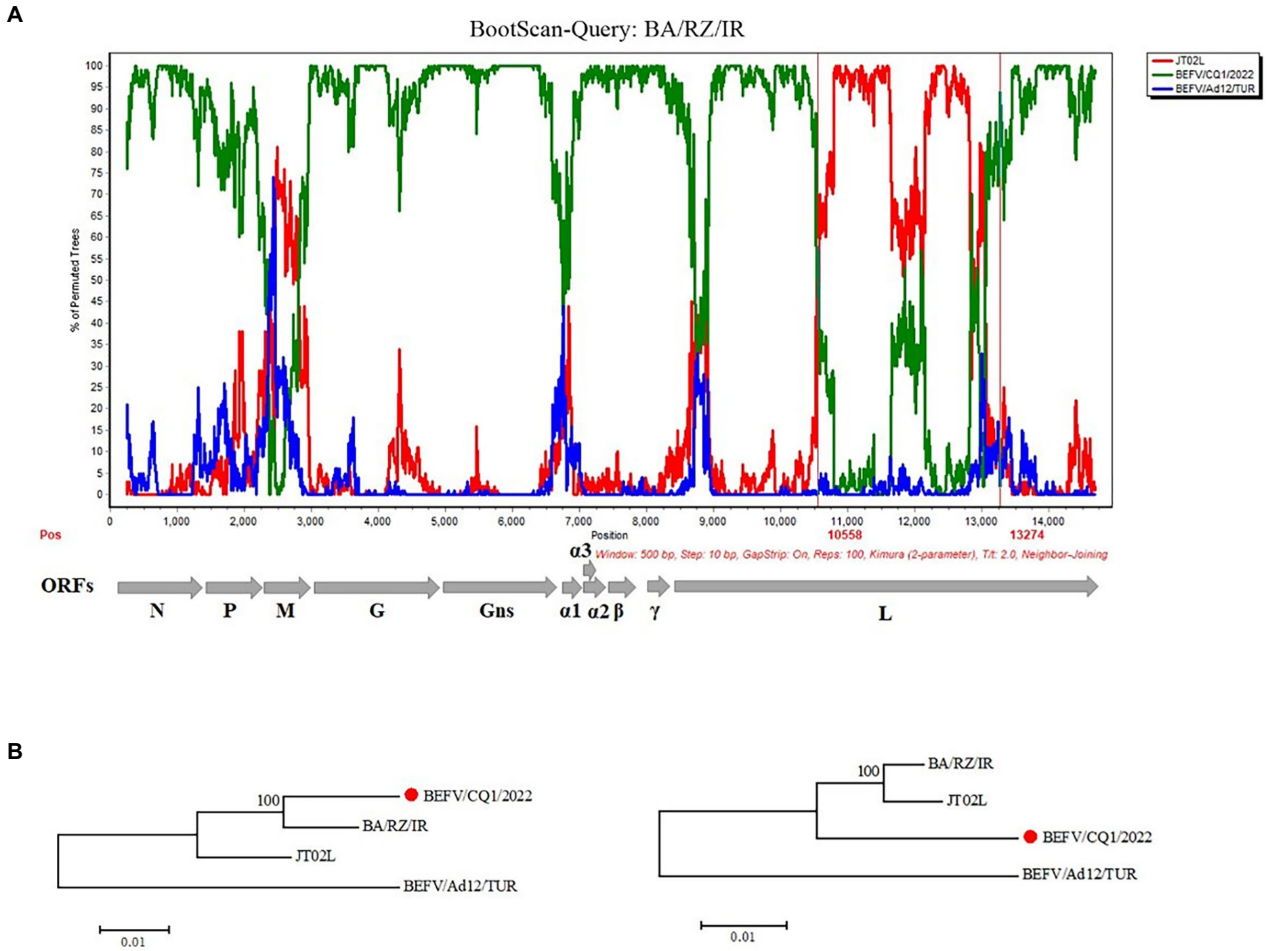
Recombination analysis of the BA/RZ/IR isolate. **(A)** BootScan analysis in the Simplot software was performed with BA/RZ/IR as the query sequence and BEFV/CQ1/2022 (green) and JT02L (red) as putative parental isolate and BEFV/Ad12/TUR (blue) as the outgroup isolate. The red vertical lines showed the potential breakpoints at position 10,558 and 13,274 nt. **(B)** Phylogenetic trees of non-recombination region (the corresponding loci in the alignment: 1–10,558; 13,275-terminal site) and recombination region (the corresponding loci in the alignment: 10,559–13,274) were shown below, respectively. The phylogenetic trees were constructed using the neighbor joining (NJ) method in MEGA 5.2 software with 1,000 bootstrap replicates.

To examine the potential recombination events in the BEFV genomes, we performed recombination analysis of the complete sequence alignment of BEFVs by the RDP and SimPlot softwares. The bootscan analysis was performed in SimPlot 3.5.1 software by the Kimura 2-parameter method, and the result showed that the BA/RZ/IR strain from Iran might be a putative recombinant. Furthermore, the BA/RZ/IR-like strain BEFV/CQ1/2022 was more likely to be the major parent-associated strain and JT02L might be the minor parent-like strain. In addition, according to the results of RDP, two putative recombination breakpoints were located at 10558 nt and 13,274 nt in the alignment, respectively; and the recombination region was located in the BEFV L gene (Figure 5A). To further validate and confirm the putative recombination event, phylogenetic analyses of the recombination and non-recombination regions separated by recombination breakpoints were performed. Then, phylogenetic tree based on the non-recombination region indicated that BEFV/CQ1/2022 and BA/RZ/IR were clustered together in a relatively independent clade, however, JT02L and BA/RZ/IR showed more close phylogenetic relationship in the phylogeny based on the recombination region (Figure 5B), which further confirmed the putative recombination event.

## 4. Discussion

Bovine ephemeral fever virus (BEFV) is the causal agent of bovine ephemeral fever and is an economically important arthropod-borne virus of cattle and water buffaloes (Walker and Klement, 2015). In recent years, outbreaks of BEFVs have been reported in several provinces in China, which have caused a considerable economic losses to the livestock industry (Kun et al., 2020). In this study, the complete G gene sequences of several BEFV samples and the whole genome of BEFV/CQ1/2022 from BEF outbreaks in Southwest China were sequenced and compared with the BEFV sequences available in the GenBank database. It showed that the G gene sequences of 4 samples from 3 different outbreaks shared 100% homology, indicating the BEF outbreaks may be caused by the same BEFV strain. Genomic sequence alignment showed that BEFV/CQ1/2022 shared high sequence identity with BEFV BA/RZ/IR from Iran and JT02L from China. However, compared to the isolates from other lineages, several hypervariable regions were found in the multi-genome alignment of BEFV/CQ1/2022 and these BEFV isolates. More interestingly, frequent initiation and termination codon mutations were found among BEFV isolates, which further led to the occurrence of several aa insertions/deletions and even the α3 ORF translation defect in BEFV isolates. However, the effect of these aa mutations on the function of corresponding viral proteins needs to be explored in the future.

Phylogenetic analysis based on the BEFV genomic sequences indicated that the BEFV/CQ1/2022 and BA/RZ/IR isolates were closely related and clustered in a sublineage of the East Asia lineage, which was consistent with the finding of a previous study that several East Asian isolates together with the Iranian isolate could construct a separate lineage (Bakhshesh and Abdollahi, 2015). Moreover, one recent study showed that BEFV could be classified into 4 phylogenetic groups (Omar et al., 2020). In this study, a phylogenetic tree based on G ectodomain encoding sequences further revealed that the global BEFV isolates were clustered geographically and separated into four phylogenetic lineages with the African and several Australian isolates clustering in a distinct lineage separate from other lineages. Additionally, the isolates from East Asia lineage were found to be more diverse and this lineage can be separated into four sublineages. Particularly, several Middle East-originated isolates from Turkey, Iran, and Egypt were found to be clustered in sublineages 2 and 4 of the East Asia lineage, indicating a close evolutionary relationship between the East Asian and Middle Eastern isolates.

Interestingly, the causative agents LYC11 and Shandong of the BEF outbreaks in China in 2011 were clustered in sublineage 2 of the East Asia lineage. Coincidentally, several Iran and Turkey-derived isolates collected in 2012–2013 were also clustered in sublineage 2, and Iran-derived isolates were closely related to the isolates LYC11 and Shandong from Mainland China. In addition, a molecular epidemiological study in Egypt reported that a BEFV isolate collected in 2005 was closely related to a 2004 isolate from Taiwan, suggesting that BEFV may have been imported through cattle trade from East Asia to the Middle East (Aziz-Boaron et al., 2012). Moreover, it had been reported that BEFV could spread *via* biological vectors through wind dispersal between East Asia and the Middle East (Chaisirirat et al., 2018). Hence, due to the relatively close geographical distance and frequent trade between East Asia and Middle East countries, more East Asia-originated isolates may have appeared and circulated in the Middle East countries. Similarly, an India-derived isolate was located in the Middle East lineage with the isolates from Middle East countries and several Australian isolates were found in the Africa lineage. The crossing of genetic relationships between isolates from different geographical origins suggests that the evolution and variation of BEFV are intensified globally, which requires extensive attention from all over the world.

The envelope glycoprotein (G) of BEFV has the capacity to induce the neutralizing immune response and then protect against experimental challenge in cattle. Neutralizing antigenic determinants of G protein have been identified as 4 independent antigenic sites (G1–G4). Furthermore, the amino acid (aa) sequences corresponding to G1, G2, and G3 have been determined. Here, the aa sequence differentiation in the antigenic sites G1-G3 of the BEFV isolates was analyzed. Overall, we found a certain amount of aa variation in the G1 to G3 antigenic sites between isolates used in this study. In the aa alignment of G protein among East Asian strains, aa variation sites were relatively scattered and the representative aa variation was not common. By contrast, consistent with the previous studies (Kato et al., 2009; Zheng and Qiu, 2012), the representative substitutions (E223D) and (T224K) in the putative glycosylation sites of G3 were found to be present in the majority of isolates in the Middle East and Australia lineage, respectively, which may play an important role in antigenic discrimination between the isolates of East Asia lineage and Middle East & Australia lineages. More representative aa variation sites, including those (N222D and E223D) in the putative glycosylation sites, were also found in the Africa lineage. It can be speculated that these altered aa sites, especially the putative glycosylation sites, were likely to affect the conformation and antigenicity of G proteins, which may challenge the effectiveness of BEFV vaccines currently in use. However, further experiments using neutralizing monoclonal antibodies are still needed to determine the precise antigenic roles of this aa variation in the corresponding BEFV isolates.

Previously, He et al. had proposed that the East Asian BEFV was emerged by the recombination event between Middle East and Australia lineages, which provided the evidence of homologous recombination as one of genetic and variation mechanisms of BEFV (He et al., 2016). In this study, we performed a recombination analysis of geographically distinct BEFVs based on the whole genome sequences and found that potential recombination events might occur between these BEFV isolates. Furthermore, BEFV BA/RZ/IR collected from Iran was identified as a putative recombinant with the Chinese strain JT02L as its minor parent-like strain. In addition, considering that the major parent-associated strain BEFV/CQ1/2022 was isolated in Southwest China in 2022 and the putative recombinant BA/RZ/IR was collected in Iran in 2013, we assume that an ancestral strain of BEFV/CQ1/2022 may be the actual major parent-like strain of BA/RZ/IR. However, due to the absence of sequence information for earlier isolates in the sublineage of BEFV/CQ1/2022, the actual major parental isolate was not accurately deduced.

## 5. Conclusion

In conclusion, we sequenced the whole genome of one BEFV isolate in Southwest China and performed a comprehensive genetic and evolution analysis with the global BEFV sequences. Genomic analyses of the BEFV genomic sequences revealed remarkable inter-isolate divergence, and the sequence divergence strictly corresponded to evolutionary relationships between the isolates. Phylogenetic analysis indicated that isolates from East Asia lineage were diverse and the current isolate BEFV/CQ1/2022 clustered within a sublineage of the East Asia lineage. Additionally, recombination analysis provided an evidence of the recombination among BEFV isolates. These findings obtained in this study will increase understanding of the epidemiology and evolution of BEFV.

## Data availability statement

The data presented in the study are deposited in the GenBank repository, accession number OP887034.

## Ethics statement

The animal study was reviewed and approved by Institutional Animal Care and Use Committee of Southwest University, Chongqing, China (IACUC-20221114-03). Written informed consent was obtained from the owners for the participation of their animals in this study.

## Author contributions

CY, YP, NL, and RF performed the conceptualization. JC, CY, and YP performed the formal analysis and funding acquisition. JC, CY, and ML wrote the manuscript. JC, ML, YL, LY, YT, RD, and MX executed experiments and analyzed the data. All authors contributed to the article and approved the submitted version.

## Funding

This research was funded by the China Agriculture Research System of MOF and MARA (Beef/Yak Cattle, CARS-37), the Fundamental Research Funds for the Central Universities (SWU-KT22016), and the Chongqing postgraduate research and innovation project in 2022 (CYB22154).

## Conflict of interest

The authors declare that the research was conducted in the absence of any commercial or financial relationships that could be construed as a potential conflict of interest.

## Publisher’s note

All claims expressed in this article are solely those of the authors and do not necessarily represent those of their affiliated organizations, or those of the publisher, the editors and the reviewers. Any product that may be evaluated in this article, or claim that may be made by its manufacturer, is not guaranteed or endorsed by the publisher.
