# Supplementary material for: Emergence and genomic analysis of a novel sublineage of bovine ephemeral fever virus in Southwest China
Source: Front Microbiol. 2023 Mar 22;14:1161287. doi: 10.3389/fmicb.2023.1161287 (PMC10073494; doi:10.3389/fmicb.2023.1161287)
Supplement: Supplementary file 1 [file Table_1.DOCX]

**Table S1 Amino acid variation between BEFV/CQ1/2022 and other representative isolates**

| GENE | Amino acid variation found in JT02L, IND/IDR/BEFV/2019, BB7721, and RSA/OBP/BEF2008 | | | |
| --- | --- | --- | --- | --- |
|  | KY315724_JT02L | MN905763_IND/IDR/BEFV/2019 | AF234533_BB7721 | MW463337_RSA/OBP/BEF2008 |
| N | R78K, H320R | E368G, | E241G, E268D, E360D, D363N, 372-373 (EN>GD) | E360G |
| P | D30E, N49S, S50N, G58E, D60G, V61I, S95T, N107D, 115-116 (LV>FT), H125N, Q149H, I158T, A166V, V178I, I186V, H255Y | D30E, 41-43 (NNK>SSA), E45G, G58E, P59A, D60G, V61I, S95T, N107D, K110R, V114I, 115-116(LV>PT), N121M, 125-126 (HQ>NK), 150-154 (IRSDV>MKLEI), G164N, A166V, R167N, K173N, 177-178 (SV>GL), 180-183 (SNKR>LKRK), D187G, E189D, G245D, K264R, I272V | K20R, D30E, N41S, S50N, I54V, G58E, D60S, V61I, I77M, L86M, S95T, D101N, N107C, 112-113(CN>YV), 115-117(LVK>SSE), H125N, N130E, Q132P, A139E, D153E, 164-165(GL>NQ), A166V, R167K, K174Q, E179K, 181-183 (NKR>KRN), 185-187 (IID>VVN), E189D, 194-197 (TECN>MKYD), V213I, Y237S, A242T, H255Y, S262R, S267N | E32D,34-35(EN>DH),T37N, 41-42 (NN>GR), E44D, S50N, 54-55(II>VV), 57-58 (QG>PE), P59T, D60G, 94-95(VS>IT), 108-110(TYK>PCR),112-116 (CNVLV>RSAYL), 119-120 (LC>VP), S123P, 125-126 (HQ>IW), 128-130 (KNN>RRD), A139V, 142-144 (AQV>EHA), R151I, D153E, 164-165 (GL>NQ), A166V, R167K, K174R, 176-177 (KS>NN), 179-181 (ESN>DLI), 183-185 (RDI>NNV), D187C, E189N, H193T, C196H, D205E, K209R, H255Y, S262G, S265N, I272T |
| M | D45E, K212R | D45E, Q52N, L220V | S10P, G23R, D37A, D45E, D77E, C169G, 221-222 (SR>VK) | 44-45 (DD>AE), D198E, |
| G | R3K, M7I, H15N, R277K, R558K, S562F, N612S, | R3K, M7I, 12-14 (NGI>RRL), K18R, E223D, N250S, R277K, Q410L, K503T, R517K, R558K, N570S, T572A, T581A, E583G, M587V, S610G, N612S | R3K, M7I, G13K, F16L, D72A, K83R, K198E, S206N, 223-224 (ET>DK), R249K, R277K, S366N, I399V, R419G, 435-436 (TK>NR), I459L, E465D, I480V, K486R, S499N, R517K, R558K, N567S, N570R, T572A, I580T, 586-587(KM>RV), S610G, N612S, 614-615 (PI>QT) | M7I, N12S, I14H, 16-17 (FE>LK), P31S, K56R, E108D, T179A, T190I, V195A, I200R, R218M, 222-223 (NE>DD), S268A, F270L, N272S, I275V, R277K, K279R, R291G, Y383H, W392L, Q410L, V447I, E465K, V496I, K503N, I506V, R558K, 585-587 (MKM>ARV), K609R, S610G, N612S, I615T, 618-619 (DR>NK) |
| GNS | I8L, T11V, T15K, A20T, I143V, V267I, G316E, A348T, R412K, V449I, K462R, N487D, D490E, F497S, R512W, S518G, T527I, K566R | I8L, T11I, Y12C, 15-16 (TT>KN), A20T, L37M, R52Q, S119N, I143V, N177S, A178P, L268M, G316E, A348T, I380V, R412K, V449I, K462R, I482V, 486-487 (KN>RE), D490G, L492S, T495N, F497S, L504V, D505E, R512W, E514K, S518R, K520I, K522N, T527I, K566R | I9V, T11I, C13G, T15R, K18Q, A20T, K38R, R52Q, S119N, I143V, V157I, K172Q, A178P, K238R, R248K, G316E, S317L, S320P, A348T, S350N, S355N, K382R, F404L, R412Q, E426H, H429Y, L442F, S447R, V449I, K462R, I482V, 486-488 (KNT>RDI), D490E, L492S, N493D, T495D, 497-498 (FS>SQ), D502K, D505E, L510W, R512W, N516T, E524D, T527R, K566R | 5-6 (LF>VL), I8L, T11I, 12-13 (YC>CY), T15Q, A20T, S32R, I49S, R52Q, E72D, A111I, E115D, S119N, K135R, I143V, K150R, K172R, A178P, I181V, Y183H, S192P, R248Q, I266L, R275K, E279A, N302S, N314T, G316E, K336R, A348T, S355N, I376V, E387D, 403-404 (DF>NL), G406S, N409S, R412K, 421-422 (DK>EE), P424S, H429Y, R437K, D440N, T443S, V449I, T452I, I466V, R472G, I482V, 486-490 (KNTED>TDKKE), L492S, 495-498 (THFS>SYSG), V500I, 505-506 (DG>ES), Y509R, 511-512 (NR>DW), 514-516 (EEN>NGE), S518I, K520E, 524-525 (EG>NK), T527R, K531I, T533I, N536I, K566R, N573S, 579-586 (QRFFKLDY>△) |
| α1 | N66T, K80R | N11D, Q15K, A55V, V62F, K80R, K83R | Q15R, N26I, L64F, K67Q, V75I, K78R, K80R | Q15R, V22I, N26D, I42V, R53K, Q60K, L64V, R74Q, K77R, K80R |
| α2 | 1-4 (MFGY>△),Y66C, H79Q, N86S, K110Q | C8S, E40D, Y66C, H79Q, D100E, K102R | C8S, N18S, A35T, K39E, V70M, H79Q, L81F, M85L, I94L, K107R, K110R | C8S, 18-19 (NR>DK), E31D, A35D, Y66C, V70I, L75S, H79Q, L81F, M85L |
| α3 | R23G, F27L, V32M, 35 (+QRELVMFGRCKETDINRVPESF) | G6R, L8F, C10R, P11L, 16-35 (KIGHNAKRRSKFRLLSVAQH>△) | G6R, L8F, C10H, P11L, 14-15 (EG>GR), I17T, A21V, 23-25 (RRS>GGG), 31-32 (SV>PM), 35 (+QRELVVSGGHEKTNIN) | Not available (due to the start codon alteration (ATG to GTG)) |
| β | I97L | N68S, P121H, I133V | R16K, I52V, 68-69 (NN>SD), D92E, 108-147 (EEYGVIDISIKVEPRGLRFLKRSSEIDICDIPRKVRVVPT>△) | 35 (+K), I52V, V55A, N68S, 84-85 (LE>SA), N88T, V94I, R140K |
| γ | R42K | A38T, R42K, H64Y, N75K | A38T, R42K, 64-65 (HV>YI), N75K | A38S, R42K, H64Y |
| L | 1-3 (MKK>△), F10L, P15T, H18N, K72R, R98K, D408G, I413V, I466V, G618S, R782S, G788V, D893E, R1096K, I1105V, S1365N, E1630G, A1664S, L1676I, I1856T, Q1870R, K1892R, S1911N, K1920R, T2038M, I2043V, Y2046D, I2055V, Y2074N | 1-3 (MKK>△), P6S,15-16 (PG>NS), H18N, C25F, R98K, I104V, V123T, E159D, D163N, K217R, D232E, S234G, D288N, H410Q, R735K, R782S, D893E, N897D, R984K, R1096K, K1102R, I1105V, Y1158S, V1324I, S1365N, T1389N, R1537K, 1566-1567 (QE>RD), M1573I, A1600V, T1628N, E1630G, 1635-1636 (KL>QM), K1638E, S1648N, A1664S, L1676I, D1769E, L1771F, R1799K, K1853R, K1920R, K1940N, L2019S, I2043T, L2047I, D2051E, K2061R, Y2074N, E2075R, Q2110K, V2135I | 1-3 (MKK>△), P6S, F10L, 15-16 (PG>HD), H18N, E22G, C25F, E28D, F30L, K65R, Y74H, G80R, T84S, 87-88 (LR>FK), I104V, V123A, E159N, D218E, L229F, I252V, I272V, D288N, H410Q, T443N, Q656R, R782N, E886Q, D893E, S945Y, R984K, L1092F, R1096K, S1104F, I1105V, E1110D, Y1158S, R1209K, N1223T, N1274S, V1324I, S1365N, E1383D, S1428Y, F1433L, R1537K, K1562R, 1609-1610 (KD>RE), L1627I, D1632G, K1635Q, D1641E, S1648D, A1664S, I1667S, L1676I, V1686I, V1708M, G1710R, R1799K, G1823D, I1856T, T1879M, K1894R, S1918P, K1920R, S1931N, K1943E, E2009K, L2019S, L2052F, L2069I, Y2074N | P6S, F10L, P15N, H18N, C25F, I33L, D35N, D37E, K72Q, E76G, Q82H, R88N, R98K, I104V, R108K, D111E, K118R, V123T, V125I, E159D, I161V, K209R, I252V, D288N, K335R, D399E, H410Q, I413V, K435R, T443N, E625D, R695K, R782S, D893E, R984K, R1096K, I1105V, E1110D, Y1158F, K1174R, I1248V, E1294D, V1324I, S1365N, L1467I, 1536-1537 (KR>RK), S1598N, A1600V, R1602K, 1626-1627 (YL>CA), E1630D, 1634-1635 (VK>IQ), E1642G, R1644Q, S1648N, A1664S, 1666-1667 (SI>NT), L1676I, I1706V, V1708T, R1799K, K1853R, I1856T, S1911N, K1920R, R1927K, T1978I, N2002S, 2038-2039 (TN>AD), I2043T, L2052M, N2058Y, Y2074N, K2077R, N2131S, V2135I, S2137T |

Note: Single amino acid residues changes were recorded in the following format, including the BEFV/CQ1/2022 reference isolate amino acid, its position, and the amino acid residue found in the other representative isolates. Insertions were indicated by the amino acid position in BEFV/CQ1/2022 followed by “+” and the new amino acid in the other representative isolates. Deletions were indicated by the symbol △. Sequential amino acid changes are shown with the BEFV/CQ1/2022 amino acid positions first, followed by the relevant BEFV/CQ1/2022 amino acid residues, then with “>”, and finally the alternative amino acid residues of the other representative isolates.
